# Ketogenic Diet Intervention for Obesity Weight-Loss- A Narrative Review, Challenges, and Open Questions

**DOI:** 10.1007/s13668-025-00634-3

**Published:** 2025-03-08

**Authors:** Adina Bachar, Ruth Birk

**Affiliations:** Nutrition Department, Health Sciences Faculty, Ariel University, Ariel, Israel

**Keywords:** Ketogenic diet, Obesity, Weight reduction, Lean body mass, Ketone bodies, Adherence

## Abstract

**Purpose of Review:**

The ketogenic diet (KD) has gained clinical attention for its potential benefits in weight loss and metabolic syndrome. By mimicking fasting through carbohydrate (CHO) restriction, KD shifts energy utilization to ketone bodies (KB) instead of glucose. Despite promising results, the effects on different weight loss indicators remain controversial, with challenges in monitoring adherence standards, optimal macronutrient composition, potential risks, and long-term sustainability. This article aims to review the different weight-loss outcomes of KD interventions for obesity, monitored by KB (adherence indication).

**Recent Findings:**

Current literature on KD interventions for obesity weight loss monitored by KB show reduction in different outcomes, including body weight, body mass index, waist circumference, visceral adipose tissue, fat mass, and body fat percentage. Minor decreases in lean body mass and skeletal muscle mass were noted without resistance training. Variability existed in adherence (KB markers), CHO intake (7–27% of daily energy), diet duration (28 days to 12 months), and follow-up frequency (weekly to biannual). KD, particularly accompanied by exercise, positively influenced appetite regulation.

**Summary:**

KD interventions improves weight-related outcomes in participants with obesity but presents challenges in lean body mass reduction without resistance training and adherence variability. Standardizing methodologies, refining interventions and suitability to sub-populations, setting KB markers, and defining clinical relevance are essential for optimizing KD effectiveness.

**Graphical Abstract:**

The physiological effect of KD

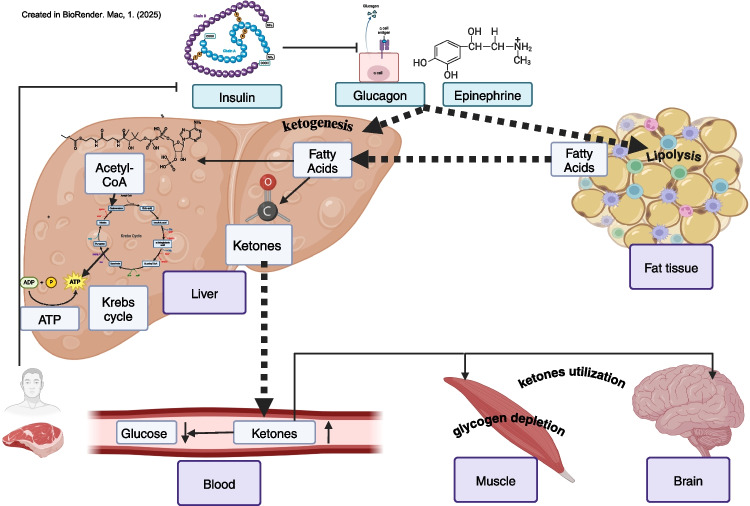

## Introduction

### What is the Ketogenic Diet (KD)?

The KD is characterized by a dietary composition that induces an elevation in blood Ketone Bodies (KBs), namely a state of nutritional ketosis [1–10]. KD mimics a condition of fasting without the negative consequences of starvation [2, 3, 6, 9–11], resulting in a shift in the body's main energy source from glucose to KBs. The initial clinical use of KD began in the early 1900s to manage epilepsy and minimize seizure activity [8, 12–14]. By inducing ketosis, patients had mitigation of seizure activity and improvements in cognitive function, highlighting the capacity for ketones to provide energy to the brain. From the 1960s onwards, very low carbohydrate KDs (VLCKD) have become a common method for obesity treatment [2, 15]. Scientific studies over the last few decades have provided evidence for the therapeutic potential of KDs in many other pathological conditions, including Type 2 Diabetes Mellitus (T2DM), polycystic ovarian syndrome (PCOS), acne, neurologic diseases (epilepsy, Alzheimer’s, stroke), cancer, and the amelioration of respiratory and cardiovascular (CVD) risk factors [1, 2, 4–6, 11, 16, 17].

Therapeutic KD, unlike any low carbohydrate diet (LCD), consists of limited carbohydrate (CHO), mostly to a maximum of 50 gr/day, high fat, and moderate protein content [2, 7, 10, 11, 13, 18, 19]. However, total CHO can be lowered to 30 gr/day to adapt more effectively to the body’s use of KBs. Concurrently, dietary fat increases to 70–80% of total calories, while protein comprises 20%. However, different protein/fat ratios are commonly adopted [2, 10]. Additionally, the KD composition consists of minimally processed food products. The KD food items frequently recommended include eggs, meat, and fish (particularly oily fish), plant oils (e.g., olive oil and coconut oil), giblets (e.g., the liver, heart, and kidneys), non-starchy vegetables (all, but primarily greens, i.e., broccoli, spinach, lettuce, arugula, and kale), avocado, olives, and nuts [10]. During periods of low CHO intake, fasting, intense exercise, starvation, or due to a complete lack of insulin in untreated type I Diabetes, glycogen depletion occurs, and blood glucose and glycogen stores are minimized. Consequently, resulting in the induced production of water-soluble KBs or ketones]e.g., Acetoacetate, Beta-Hydroxy- Butyrate (BHB), and Acetone], by the liver from fatty acids (FA) as a source of energy [9, 16, 17]. The hormonal activation of lipolysis and ketogenesis is mediated by epinephrine and glucagon and opposed by insulin. glucagon stimulates adipose tissue lipolysis to release stored FA, which is routed to the liver and stimulates liver glycogenolysis to restore blood glucose. In addition to forming ketones, FA can be converted to acetyl CoA—an intermediary substrate between FA oxidation and the metabolism of glucose that enters the citric acid cycle and then undergoes oxidative phosphorylation for ATP generation [9, 16], (Graphical abstract). Conversely, in response to high blood glucose (i.e., after a high CHO meal), insulin levels rise and shut off ketogenesis in favor of De Novo lipogenesis (DNL) and fat storage. Thus, ketosis represents a metabolic shift from an insulin-dependent glucose-based energy system to an enhanced capacity to utilize dietary and stored fat as fuel [2, 3, 5, 6, 9, 11, 14, 16, 20–24]. This process generates fat-derived energy in the liver and then shipped throughout the body to supply energy to the renal cortex, heart, and skeletal muscles [3, 4, 25, 26]. Furthermore, ketones can cross the blood–brain barrier and replace glucose as the primary energy source for the brain [27]. Ketones can provide up to 60% of ATP required by the body; the remainder is derived from endogenous gluconeogenesis that utilizes glycerol from triglycerides (TG) and glucogenic amino acids from protein for glucose production. Nutritional ketosis can be defined as the intentional restriction of dietary CHO intake to accelerate the production of ketones and induce a metabolic effect that stabilizes blood sugar, minimizes insulin release, and thereby mitigates the downstream anabolic and tumorigenic effects of longstanding insulin resistance (IR). The hallmark of nutritional ketosis is blood ketone (BK) levels of 0.5 to 3 mm/L [2, 7, 10, 15, 16, 23]. This contrasts with, and should not be confused with, the pathophysiologic state of type 1 diabetic ketoacidosis (DKA), where ketone levels are 5–tenfold higher. Additionally, while the body maintains normal pH and blood glucose levels in nutritional ketosis, DKA is associated with extremely elevated blood glucose and acidic PH [2, 12, 15, 16, 23].

### Different Compositions of KD

A broad spectrum of KD definitions and macronutrient compositions exist, with some being “stricter” than others [2, 6]. Overall, the KD reverses the typical dietary pyramid use of macronutrients, promoting a restrained CHO intake and more liberal consumption of proteins and fats [5]. The four main KD types are: 1. The classic KD is high in fat- 90% of the total energy intake (TEI), and low in CHO and proteins. The ratio (gram) of fat to CHO and protein is 4:1. Classic KD is a strict diet, that needs to be closely monitored. 2. The medium-chain TG (MCT) diet allows for more generous intakes of protein and CHO and uses MCT oil as a source of dietary fat. 3. The modified Atkins diet (MAD) is freestyle, allowing for a free intake of fat, protein, energy, and fluids, while limiting CHO intake to 10–15 gr/day in the first month, subsequently increasing to 20 gr/day. 4. A low glycemic index (LGI) diet, based on KD glucose-lowering effect. LGI permits 10% of TEI to come from foods with an LGI (below 50) [2, 4, 9]. Beyond the 4 main KD types, other popular dietary compositions restricting CHO exist, such as the LCD, which restricts CHO to < 130 gr/day. However, not all LCDs induce nutritional ketosis, which occurs only when the intake of CHO is ≤ 50 gr/day [5, 6, 24, 28]. Thus, special attention should be given to the definition of the diet. Furthermore, any lack of demonstrable favorable effects following non-KDs should not be extrapolated to that of KDs, as other dietary approaches significantly differ in their metabolic and physiological impacts [6, 28]. This review focuses on KD without or with a moderate calorie restriction as a nutritional treatment for obesity weight loss.

### Obesity and KD

The KD has regained attention as medical nutrition therapy (MNT) for overweight, obesity, Metabolic syndrome, and T2DM [2–4, 11, 13, 19, 21, 28–30]. These conditions are often linked to CHO intolerance and IR, suggesting that reducing dietary CHO intake may improve metabolic health [5]. Some studies have shown significant benefits of adhering to restricted CHO intake (≤ 50 gr/day) even for relatively short durations, such as six months [7].

These findings highlight the potential of the KD as a viable approach for managing obesity and related disorders, warranting further investigation into its long-term effectiveness and safety. Emerging evidence suggests that KD can be considered a first-line MNT for obesity management due to its ability to suppress hunger, reduce lipogenesis, increase lipolysis, enhance metabolic efficiency for fat utilization, and boost energy expenditure [1].

There are several suggested mechanisms through which KD may improve weight management, in brief:

- 1. Reduction in energy intake [4, 11]. Various factors contribute to this reduction, such as:
  • Increased energy availability during the late post-prandial period which helps sustain energy levels [5, 23],
  • Improved food quality in KD, characterized by nutrient-dense, low-glycemic, and satiating foods, leads to a natural reduction in energy intake by prolonging fullness and stabilizing blood sugar levels [23, 31],
  • Hormonal changes that impact hunger, including reduced ghrelin, and increased levels of glucagon-like peptide-1 (GLP1), adiponectin, and cholecystokinin (CCK), all of which contribute to a decreased perception of hunger [2, 3, 5, 6, 9, 11, 13, 14, 16, 21, 23, 31, 32].
  • Nutritional ketosis itself, where KBs exert anorexigenic effects, reducing appetite and promoting satiety [3, 6, 9, 12, 14, 16, 20, 33, 34].
  • Satiety driven by the higher protein intake typically associated with KD, which helps to prolong feelings of fullness [13, 16, 23, 32].
- 2. Reduction in highly processed, or “ultra-processed” food consumption.
  - KD naturally limits the intake of Ultra-processed foods which are energy-dense and nutrient-poor. These foods, often high in sugar, salt, and fats, are linked to increased energy consumption and weight gain [23, 35, 36].
- 3. Insulin reduction and improved metabolic regulation that contributes to:
  • Down-regulation of anabolic pathways [3, 9, 11, 29, 29, 31, 37].
  • Promotion of fat oxidation and depletion of adipose tissue stores, leading to weight loss [37].
  • Alleviation of IR through reduced external insulin requirements and secretion [3, 6, 9, 11, 16, 22, 28, 29, 37]
  • Direct insulin-sensitizing effects of the KD due to its low CHO content [5, 6, 16].
- 4. Enhancing fat oxidation and energy expenditure.
  • KD increases fat oxidation, lowers the respiratory quotient (RQ), and reduces visceral adipose tissue (VAT) [9, 16, 31].

### Limitations and Challenges

Despite its therapeutic potential, there is a lack of longitudinal data on the long-term effects of KD. Concerns have been raised about the potential negative impact of long-term low-CHO, high-fat KD practices, on adherence, and the risk of CVD, especially when evaluated through the lens of the traditional high-CHO, low-fat atherogenic model [2, 6, 38]. However, emerging research increasingly challenges the long-standing recommendations of low-fat, high-CHO for CVD prevention, promoting a reassessment of current global dietary guidelines. Nevertheless, studies specifically examining the health effects of very high-fat consumption over the long term remain limited, leaving critical gaps in understanding its impact on overall health (5].

One commonly cited limitation of KDs is their potentially low micronutrient content, which could lead to nutrient deficiencies if not managed properly [5–7, 23, 39]. For instance, while thiamine deficiency has been reported in some cases, other consistent deficiencies have not been widely observed in the literature. To reduce this risk, it is typically advised to carefully plan the diet, incorporating nutrient-dense low-carb vegetables, fortified foods, or supplements to ensure sufficient intake of essential micronutrients [2, 5, 23].

Concerns have been raised about muscle mass and bone health in individuals following KDs, especially for those engaging in prolonged CHO restriction. The reduced availability of CHO may impact muscle glycogen stores and exercise performance, potentially influencing muscle maintenance. To address this, ensuring sufficient protein intake and engaging in regular resistance training can help preserve muscle mass on a KD [5, 6, 40]. Vitamin D metabolism and bone health are additional areas of concern. The limited intake of vitamin D-rich foods on a KD, along with the ketogenic state itself, may affect calcium and bone metabolism. To mitigate these challenges, incorporating vitamin D supplements, consuming calcium-rich ketogenic-friendly foods, and monitoring bone health markers can help address these concerns effectively. These preventive measures may alleviate many of the diet's potential limitations [5].

While some studies suggest that KDs can provide short-term benefits for managing T2DM and obesity, questions about their long-term safety remain. For example, research on the potential impact of KDs on renal function is limited, especially given the higher protein intake typically associated with the diet. This raises concerns about kidney stress or damage, particularly for individuals with pre-existing kidney conditions. Regular monitoring of renal markers and adjusting protein intake according to individual health status can help reduce these risks [5, 7]. Another area that requires further investigation is the effect of KDs on gut microbiota. The diet's low fiber content and altered macronutrient ratios may influence microbial diversity and gut health. Changes in microbiota composition could affect inflammation, digestion, and metabolic health. To support gut health on a KD, including fiber-rich, low-carb foods and considering probiotic supplementation may be beneficial [5, 7].

Endocrine changes are also a potential concern, as the restrictive nature of KDs and altered hormonal environments may affect reproductive health, thyroid function, and other hormonal systems. These potential effects highlight the need for individualized monitoring and diet modifications, ensuring the KD aligns with both short-term objectives and long-term health outcomes. Further research is needed to better understand and address these issues [7, 41].

KDs are not recommended for certain special conditions and populations [42]. For instance, children following such a strict diet may experience developmental issues, fatigue, and an increased long-term risk of CVD disease [2, 6]. Animal studies suggest that elevated ketone concentrations during pregnancy could have harmful effects on both the fetus and mother [2, 6]. However, the precise pathophysiological mechanisms remain unclear [2]. KDs are also challenging for type 1 diabetes patients due to the risk of hypoglycemia and ketoacidosis [2, 6]. There are concerns about KDs in individuals with kidney disease due to the high protein content, which could worsen kidney function decline and increase the risk of kidney stones from higher urinary calcium and reduced citrate levels. A vegetarian KD may offer a safer alternative, reducing the acid load and improving kidney health outcomes. For those with chronic kidney disease, individualized dietary planning is essential [43]. Additionally, KDs can lead to an increase in uric acid levels, especially during the initial phase when rapid weight loss and heightened ketone production occur. While this rise in uric acid raises concerns about gout, an attack is not always triggered. After the adaptation phase, gout attacks tend to decrease as uric acid levels stabilize. Maintaining proper hydration and monitoring uric acid levels can help minimize the risk, and the body's adaptation to the ketogenic state may ultimately reduce the frequency of gout flare-ups [44].

### Aim

This narrative review focuses on the effects of KD weight-loss interventions monitored by KB on weight-related indicators, including body mass index (BMI), body weight (BW), fat mass (FM), waist circumference (WC), lean body mass (LBM), waist-to-hip ratio (WHR), visceral adipose tissue (VAT), body fat percentage (BFP), and appetite in individuals with obesity.

### Methods

A literature search was conducted on PubMed. Inclusion criteria were clinical, and intervention trials published in English over the past five years, involving adult human participants with obesity implementing a KD for weight loss and reporting KB. Search terms included “obesity” OR “overweight” AND “Diet, Ketogenic” OR “ketogenic diet” OR “keto diet” OR “very low carbohydrate diet” OR “low carbohydrate diet” OR "Low-calorie ketogenic diet". Exclusion criteria included studies involving children, adolescents, type 1 diabetes, pregnancy, non-ketogenic or very low-calorie interventions, bariatric surgery, or trials that did not measure KBs or used ketone supplements (Table 1)**.**

**Table 1:** Inclusion criteria

Criterion	Description
Population	Adult human participants with obesity
Concept	Studies exploring the effects of KD interventions on weight-related indicators (such as BW, FM, and WC) in individuals with obesity. studies involving children, adolescents, type 1 diabetes, pregnancy, non-ketogenic or very low-calorie interventions, bariatric surgery, or trials that did not measure KBs or used ketone supplements were not considered
Context	Any setting where adults with obesity participated in a KD intervention study aiming to reduce weight, and reporting KB. Studies aimed to treat other chronic nutrition related diseases were not considered
Types of Evidence Source	Peer-reviewed primary intervention research studies, full-text available in English, studies published between 2019–2024, including qualitative and quantitative study designs

## Results

Following inclusion and exclusion criteria, 16 out of 156 were included (four single-arm with no control group trials, and 12 randomized trials with 2–4 control groups).

Table 2 summarizes the included studies.

**Table 2:** Summary of studies

Study population	Study design and duration	Diet/intervention	Methods used to estimate compliance and outcomes	Major outcomes	Author, year
**no control group/single-arm studies**					
***Participants***: Fourteen females with PCOS^a^ ***Age***:18—45y, ***Average BMI***^***b***^: 28.8 ± 2.1 kg/m^2^	Single-arm study 12 weeks	Ketogenic Mediterranean diet with phytoextracts (KEMEPHY) intervention ***Diet Composition (%EI***^***c***^*):* Protein: 24.1 ± 5.6% Fat: 71.1 ± 9.3% CHO: 4.8 ± 1.2% 1670 ± 90 kcal/day	Anthropometric parameters—pre-and post-intervention BK^d^—by the research team every other day for the first 6 days, then every 6 days	• Significant*** average reduction from baseline in: ***BW***^***e***^*—*9.43 ± kg ***BMI***^***f***^—3.4 kg/m^2^ ***FBM***^***g***^- 8.3kg ***VAT***^***h***^ −639.6 g ***WC***^***i***^*—*4 cm ***LBM***^***j***^**—**0.98 kg *Significant*** average increase from baseline in: ***% LBM***—7	Paoli et al.,2020 [12]
***Participants***: Thirty-five sedentary obese adults (71.4% females, 28.6% males) ***Average Age***: 37 ± 7 y ***Average BMI***: 36.1 ± 5.6 kg/m^2^	Single-arm study 12 weeks	KD^k^ intervention ***Diet Composition (%EI)****:* Protein: 20% Fat: ≥ 75% CHO: 5–10% first 2 weeks—1200—1500 kcal/day ≥ 2 weeks, no EI restriction. Average energy consumption—1365 kcal/day	Anthropometric parameters—baseline + at 1, 2, 4, 8, and 12 weeks Serum ketones—baseline + at 1, 2, 4, 8, and 12 weeks, by the team 3-day food records – baseline + at 1, 2, 4, 8, and 12 weeks	• Significant*** average reduction from baseline in: ***BW*** males—18 ± 9 kg*** females—11 ± 3 kg ***FFM***^***l***^ males—6 ± 2 kg*** females—3 ± 1.5 kg	Mohorko et al., 2019 [13]
***Participants***: Twenty-one adults (62% females, 33.3% males, 4.5% non-binary) with schizophrenia or bipolar disorder ***Average Age***: 43.4 ± 15.6 y ***Average BMI***: 34 ± 4.9 kg/m^2^	Single–arm clinical trial 4 months	KD intervention ***Diet Composition (%EI)****:* Protein: 30% Fat: 60% CHO: 10% ≥ 1200 kcal/day	Anthropometric parameters −10 times (weekly in month 1, every two weeks in months 2 and 3, and once in month 4) BK-Self-measured/ once a week	• Significant*** average reduction from baseline in: BW, BMI***—10.2 ± 5.6%, WC***—11.2 ± 7.8%, ***FMI***^m***^—17 ± 14.8%, VAT***—27.2 ± 24.7% ***SMM***^n^*—3 ± 9%	Sethi et al., 2024 [27]
***Participants***: Seventy-five adults (71% females, 27% males, 2% nonbinary) ***Average Age***: 42 ± 11 y ***Average BMI***: 33.5 ± 4.7 kg/m^2^	mHealth-based Secondary analysis of a Randomized clinical trial 24 weeks	Mediterranean-style KD Intervention with a personalized adjusted app ***Average macronutrient consumption (%EI)****:* Proprotein: 24.33% Fat: 51.7% CHO: 22.7% 1498 kcal/day	Anthropometric parameters -Participants used a Bluetooth scale for daily weight Breathe acetone—Self-measured 3 times/day For acetone scores of 0—3 or ≥ 4 mmol/l, participants were instructed to reduce or maintain their CHO intake	• At 12 weeks significant* average reduction from baseline in: ***BW***—5.6 ± 4.5 kg (5.8 ± 4.5%) ***BMI***—1.9 ± 1.5 kg/m^2^ • At 24 weeks significant* average reduction from baseline in: ***BW***- 8.5 ± 6.4 kg (8.7 ± 6.9%) ***BMI***—3 ± 2.3 kg/m^2^ Ketone levels were significantly* and positively correlated with weight loss	Falkenhain et al., 2022 [26]
***With control groups/* ≥ *2 groups***					
***With physical activity***					
***Participants***: One hundred and four adults (41.3% females, 58.7% males) ***Age***: 18- 60 y ***BMI***: ≥ 30.0 kg/m^2^, or ≥ 28.0 kg/m^2^ + one or more comorbidities (HTN, dyslipidemia, sleep apnea, or impaired glucose tolerance)	An open-label prospective weight-loss intervention study 12 weeks	Two diets: 1. Lifestyle modification (LM)- hypocaloric balanced diet (HBD, n = 57) ***Diet composition (%EI)****:* Protein: 24% Fat: 37% CHO: 39% 2. Multiphase-modified KD (MMKD, n = 22) -two cycles of 2 weeks of KD, 2 weeks of transition diet (29% EI CHO), and 2 weeks of HBD ***Diet composition (%EI)****:* Protein: 32% Fat: 41% CHO: 27% (≤ 50 g/day of digestible CHO) EI was calculated according to the measured basal metabolic rate *25 participants received Beinaglutide	Anthropometric parameters: visits 1, 3, and 6 Urinary ketones—self-measured and reported every morning	• Significant average reduction from baseline in: ***BW*** MMKD group—7 kg*** LM group—3.2 kg ***BMI*** MMKD group—2.5 kg/m^2^*** LM group—1.1 kg/m^2^ ***WC*** MMKD group—6.8 cm** LM group—4 cm ***HC***^***o***^ MMKD group—4.7 cm*** LM group—1.7 cm ***WHR*** MMKD group—0.02*** LM group—0.03 ***Fat Mass*** MMKD group—6.2 kg** LM group—3.1 kg ***BFP***^***p***^ MMKD group—4.5%* LM group—2.4% ***VFA***^*q*^ MMKD group—31.6 cm^2**^ LM group—13.4 cm^2^ ***SMM*** MMKD group—0.9 kg	Wu et al., 2022 [46]
***Participants***: Thirty-six overweight or obese male students ***Average Age***: 20.7 ± 1.4 y ***Average BMI***:31.1 ± 4 kg/m^2^	Randomized control trial 6 weeks	Three groups: 1. KD (n = 12) 1980 kcal/day Cyclical KD plan: KD for 6 days + 1 day of an ordinary diet (regular eating patterns before the study)/week A dietitian provided a nutrient list categorized into three groups: (1) encouraged to eat, (2) eat in moderation, and (3) foods to avoid 2. KD + aerobic training = AT-KD (n = 12) 2024 kcal/day 3. 3. KD + resistance training 4. = RT-KD(n = 12) 1982 kcal/day ***Average macronutrient consumption (%EI) for 3 groups:*** Proprotein: 25% Fat: 65% CHO: 10%	Anthropometric parameters- baseline and at 6 weeks Urinary ketones—self-measured weekly Participants with urinary ketone levels < 4 mmol/dL were excluded from the study The participant’s self-reported dietary daily intake via social media and monitored online by the research team	• Significant average reduction from baseline in: BW** AT-KD group—8.1 kg RT-KD group—5.7 kg KD group—5.2 kg LBM** AT-KD group—1.7 kg KD group—1.3 kg • Significant** average increase from baseline in LBM only in RT-KD group—2.7 kg	Valinejad and Khodaei., 2022 [14]
***Participants***: Fifty-eight females ***Average Age***: 21.2 ± 3.3 y ***Average BMI***: 25.1 ± 2.8 kg/m^2^	Four-arm intervention trial 28 days	Four diets + lifestyle: 1. Control group (CON, n = 15), ***Diet Composition (%EI):*** Protein: 15.9% Fat: 40.2 ± 5.7% CHO: 43.1 ± 7.9% 1990 ± 345 kcal 2. Low CHO diet (LC-CON, n = 15) 1776 ± 284 kcal/day 3. LC + high intensity interval training group (LC-HIIT, n = 15) 1871 ± 246 kcal/day 4. LC + moderate-intensity continuous training group (LC-MICT, n = 13) 2028 ± 284 kcal/day ***Average macronutrient consumption (%EI) for 3 LC groups:*** Proprotein: 25% Fat: 65% CHO: 10% * Some of the food products were provided to LC groups	Anthropometric parameters—once a week Urinary ketone—self-measured daily 3-day food records, 2 weeks before intervention and 4 times during the intervention	• Significant average reduction from baseline only in LC groups, but not in the CON group in: BW*** LC-CON group—2.9 kg LC-HIT group**—**2.9 kg LC-MICH group—2.6 kg BMI*** LC-CON group—1.1 ± 0.6 kg/m^2^ LC-HIT group**—**1.1 ± 0.4 kg/m^2^ LC-MICH group—1 ± 0.5 kg/m^2^ WC* LC-CON group—4 cm LC-HIT group**—**3.8 cm LC-MICH group—1.8 cm HC** LC-CON group—2.5 cm LC-HIT group**—**1.8 cm LC-MICH group—3.3 cm ***WHR***^r**^- 0.02 for all 3 LC groups Incorporated exercise training had no additional effects on weight loss	Sun et al., 2019^†^ [45]
***Participants***: Eighteen females with PCOS and liver dysfunction ***Age***: 18—50 y, ***BMI***: 28 −32 kg/m^2^	Randomized, open-label, parallel-group, controlled pilot trial 12 weeks	Two groups: 1. KD (n = 8) *D****iet composition (%EI)****:* Protein: 18- 27% Fat: 70%—75% CHO: 5—10% 1300—1500 kcal/day 2. Control (n = 10)—conventional pharmacological treatment (Essentiale plus Yasmin). No special diet - All participants were required to perform resistance exercises (15 min each time) × 3/week	Anthropometric parameters- baseline, and at weeks 4, and 12 Urinary ketones—Self-measured daily	• Significant** average reduction from baseline only in the KD group in: ***BW***—11.8 kg ***BMI***—4.5 kg/m^2^ ***Body Fat***—23.2 kg ***BFP***—6.3% ***VFA***—31.1 cm^2^ ***WHR***—0.05	Li. et al., 2021 [48]
***Participants***: Sixty-four adults with obesity, (57% females, 43% males) ***Average Age***: 35 ± 9 y ***Average BMI***: 30.3 ± 3 kg/m^2^	randomized controlled trial 8 weeks	Two diets: 1.Experimental -structured exercise program + low-carbohydrate meals (EX-LC, n = 33) *Foods were provided and did not exceed 50 gr CHO/day 2. control—structured exercise program + standard dietary advice (EX-CO, n = 31) *Both groups received aerobic and resistance training (4 sessions per week/45 min per session) *Neither diet included a specific calorie or energy goal	Anthropometric parameters—pre-and post-intervention BK- by the research team- pre- and post-intervention 24-h dietary recall, daily	• Significant average reduction from baseline after 8 weeks in: ***BW*** EX-LC group—4.4 ± 4 kg** EX-CO—1.8 ± 2.5 kg ***LMM***^***s***^ EX-LC group—854 ± 1670 cm^3^* ***FMI*** EX-LC group – 1.1 ± 0.9 kg. m^−2^** EX-CO – 0.6 ± 0.7 kg. m^−2^ *Significant average increase from baseline after 8 weeks in: ***LMM*** EX-CO group—143 ± 976 cm^3^* Reaching a ketogenic state (BHB^t^ ≥ 0.3 mmol/L) was associated with a significant decrease in total body fat (%,) * VAT*, FMI**, and LMM* • Significant*** average reduction from baseline after 8 weeks in: Two groups together (no significant differences between groups) ***BFP***—2.4 ± 5.7% ***VAT*—**15 ± 24 cm^3^	Perissiou et al., 2020 [40]
***Without physical activity***					
***Participants***: Forty-two adults (16.7% females, 83.3% males) ***Average Age***: 28–65 y ***Median BMI***: 28.4 kg/m^2^	Single-center, comparative, two-arm, randomized, open-label trial 2 months	Two diets: 1. Low-CHO diet (LCD, n = 21), ***Diet composition (%EI)****:* Protein: 27% Fat: 47% CHO: 26% 2. Very Low-CHO diet (VLCD, n = 21) ***Diet composition (%EI)****:* Protein: 27% Fat: 61% CHO: 12% (or 50 gr') EI was individually calculated based on ideal weight and physical activity level • *Foods were provided	Anthropometric parameters—baseline + at week 4, and week 8 Serum KB -by the team baseline + at week 8 Participants reported all food to their registered dietitian via an original smartphone app	• Significant* average reduction from baseline in: ***BW*** LCD group—5.4 kg VLCD group—8 kg ***BMI*** LCD group—2.1 kg/m^2^ VLCD group—2.6 kg/m^2^ ***Body Fat*** LCD group—2.6% VLCD group—2.8% ***WC*** LCD group—6.8 cm VLCD group—8.5 cm	Kikuchi et al., 2023 [24]
**Participants**: Two hundred obese patients (71% females and 29% males) ***Average Age***: 41.3 ± 11 y ***Average BMI***: 45.5 ± 8.5 kg/m^2^	A prospective cohort study 6 months	Two diets (based on patient preference): 1. very low-carb diet, (LCKD, n = 100) ***Diet Composition***: Protein: no goal Fat: no goal CHO: 20–50 gr no limitation of calories *After losing 10–20 kg, 5gr CHO/day added and continuation of net CHO consumption to enable weight maintenance and appetite control 2. low caloric low-fat diet, (LCLF, n = 100) ***Diet Composition (%EI)****:* Protein: 15–20% Fat: 30–35% CHO: 45% • *a reduction of 500 kcal from the total energy need	Anthropometric parameters -first visit and at six months Urinary ketones/once (at 2 weeks/4 weeks) by team	• Significant average reduction from baseline in: ***BW*** LCKD group—13 ± 10.7 kg^***^ LCLF group—4.7 ± 6.4 kg ***BMI*** LCKD group—5.1 kg/m^2***^ LCLF group – 1.9 kg/m^2^ ***Fat mass*** LCKD group—4%^***^ LCLF group—1.3% ***Visceral fat*** LCKD group—2.5 l*** LCLF group—1.2 l ***Muscle mass*** LCKD group—2.4 kg* LCLF group—0.8 kg * A significant difference in all parameters was found between the two groups Weight reduction was not significantly different between males (11 ± 12 kg) and females (8 ± 8.5 kg)	Al Aamri et al., 2022 [8]
***Participants***: One hundred and sixteen participants, (71% females, 29% males) ***Average age***: 41 y ***Average BMI***: 34 kg/m^2^	Randomized control trial 24 weeks	• Two diets: 1. Mediterranean-style KD with personalized adjusted app (n = 60) ***Average calorie and macronutrient (%EI) consumption****:* Protein: 22.8% Fat: 48.8% CHO: 27.3% 1664 kcal/day 2. A calorie-restricted, low-fat diet (LFD) compatible with standard weight loss diets and available through an app (n = 56) ***Average calorie and macronutrient (%EI) consumption****:* Protein: 20.5% Fat: 38.3% CHO: 39.5% 1590 kcal/day	Anthropometric parameters- Daily self-weight recorded in an app Breath acetone -self-measured three times/day *Participants were instructed to reduce or maintain CHO intake for scores of 0—3 or ≥ 4 respectively 24-h dietary recall, daily	• Significant average reduction from baseline after 12 weeks in: ***BW*** KD group—5.6 ± 1.1 kg*** LFD—2.5 ± 1.1 kg Significant average reduction from baseline after 24 weeks in: ***BW*** KD group—8.4 ± 1.8 kg*** LFD—2.9 ± 2.1 kg	Falkenhain et al., 2021 [25]
***Participants***: seventy-six participants with MetS, (77% females, 23% males) ***Average Age***: 40.3y ***Average BMI***: 31.8 kg/m^2^	randomized clinical trial 52 weeks	Three diets: 1. Asian KD with increased whole egg intake (Yolk-AKD, n = 28) ***Diet composition (%EI):*** Protein: ad libitum (≥ 3 whole eggs/day) Fat: ad libitum CHO: < 50 gr/day ***Average calorie and macronutrient (%EI) consumption****:* Protein: 30.8% Fat: 45.6% CHO: 23.7% 1330 kcal/day 2. yolk-free KD with egg white supplementation (White—AKD, n = 26) ***Diet composition (%EI):*** Protein: ad libitum (≥ 200 gr' egg whites/day) Fat: ad libitum CHO: < 50 gr/day ***Average calorie and macronutrient (%EI) consumption***: Protein: 28.7% Fat: 48.4% CHO: 23.6% 1276 kcal/day 3. a balanced low-calorie diet (BLC, n = 22) ***Diet composition (%EI):*** Protein: 15–20% Fat: 35% CHO: 50–60% *a reduction of 500 kcal from the total energy expenditure ***Average calorie and macronutrient (%EI) consumption****:* Protein: 18.7% Fat: 35.25% CHO: 46.1% 1192 kcal/day *Following the initial 12-week intervention period, participants were encouraged to maintain their respective dietary interventions without strict guidance for ≤ 52 wk	Anthropometric parameters- screening (week −4) + , at weeks 0, 6, 12, 35, and 52 Serum ketones—baseline + at weeks 6, 12, 35, 52, by the team 3-day food records 10 times during the intervention and follow-up	• Significant* average reduction from baseline after 52 weeks (only for AKD groups) in: ***BW*** Yolk—AKD group—4.1 kg White—AKD group—4 kg ***WC*** Yolk—AKD group—3.8 cm White—AKD group—4.2 cm *No significant difference between the two groups	Pinsawas et al., 2024 [30]
***Participants****:* forty-four adults (86% females, 14% males) ***Average Age***: 36.9 ± 10.7 y ***Average BMI***: 34.8 ± 4.2 kg/m^2^	Randomized control trial 8 weeks	Four diets: 1. calorie restriction (CR, n = 11) ***Diet composition (%EI):*** Protein: 25—35% Fat: 20—30% CHO: 45—55% * a restriction of 500 kcal from the caloric calculation of their usual diet 2. intermittent fasting (IF, n = 11) ***Diet composition (%EI):*** Protein: 25—35% Fat: 20—30% CHO: 45—55% 16/8 (fasting/eating) * a restriction of 500 kcal from the caloric calculation of their usual diet 3. KD, n = 11 ***Diet composition (%EI):*** Protein: 15–25% Fat: 70—80% CHO: 5—10% * a restriction of 500 kcal from the caloric calculation of their usual diet 4. ad libitum habitual diet (AL, n = 11), no nutritional indication	Anthropometric parameters—baseline, + at weeks 4, 5 and 8 Urinary ketone—by the research team at weeks 4, 5 and 8 24-h dietary recall, + a 3-day food record	• Significant average reduction from baseline after 8 weeks in: BW*** CR group—3.2 kg IF group—3.8 kg KD group—5.2 kg BMI*** CR group—1.3 kg/m^2^ IF group—1.5 kg/m^2^ KD group—2 kg/m^2^ VFA** CR group—8 cm^2^ IF group—9 cm^2^ KD group—16 cm^2^ BFP* CR group—1.8% IF group—1.3% KD group—3.2% WC** CR group – 4 cm IF group – 6.4 cm KD group—5.3 cm Significant average increase from baseline after 8 weeks in: SMM (%) * CR group—2.3% IF group—0.7% KD group—0.6%	Guevara-Cruz et el., 2024 [47]
***Participants:*** Ninety-one adult females ***Average Age***: 41.5 y ***Average BMI***: 32.9 kg/m^**2**^	Randomized control trial 12 weeks	Two diets: 1. LCKD, n = 46 ***Average calorie and macronutrient (%EI) consumption***: Protein: 20% Fat: 72% CHO: 8% 1810 ± 173 kcal/day 2. Control, n = 45 ***Average calorie and macronutrient (%EI) consumption****:* Protein: 18% Fat: 32% CHO: 50% 2170 ± 211 kcal/day	Anthropometric parameters—baseline + every 4 weeks BK or urinary ketone -self-measured once/week Serum ketones by the research team before and after the intervention a 3-day food record	• Significant* average reduction from baseline after 12 weeks in (only for LCKD group): ***BW*** −13.7 kg ***BMI***—5.1 kg/m^2^ ***WC***—13.7 cm ***HC***—11.6 cm ***TC***^***t***^—7.7 cm	Michalczyk et al., 2020 [19]
***Participants*:** Eleven participants (54.5% females, 45.5% males) ***Average Age***: 56.6 ± 5.8 y ***Average BMI***: 32.5 ± 4.1 kg/m^**2**^	Randomized controlled crossover trial 3 weeks	Two diets: 1. KD, n = 11 ***Diet composition (%EI):*** Protein: 20% Fat: 75% CHO: 5% EI is not reported 2. Standard diet, (SDD, n = 11 ***Diet composition (%EI):*** Protein: 10–20% Fat: 25–40% CHO: 45–60% EI is not reported	Anthropometric parameters—baseline + at 3 weeks BK- by research team, twice/day	• Significant average reduction from baseline after 3 weeks in: ***BW*** KD group—2.7 kg* SDD group—0.5 kg ***BMI*** KD group—0.9 kg/m^2^* SDD group—0.2 kg/m^2^ ***FM*** KD group—1.2 kg* SDD group—0.1 kg ***LM*** KD group—1.4 kg*	Loung et al., 2024 [17]

^†^ We included this study, even though the participants were not obese due to its focus on physical activity

^a^ PCOS- polycystic ovarian syndrome, ^b^ BMI- body mass index%, ^c^ EI- % of energy intake, ^d^ CHO- carbohydrates, ^e^ BK- Blood ketones, ^f^ BW- body weight, ^g^ FBM- fat body mass, ^h^ VAT- visceral adipose tissue**,** ^i^ WC- waist circumference, ^j^ LBM- lean body mass, ^k^ KD- ketogenic diet, ^l^ FFM-free fat mass, ^m^ FMI- fat mass index, ^n^ SMM- skeletal muscle mass, ^o^ HC- hip circumference, ^p^ BFP- body fat percentage,

^q^ VFA- visceral fat area, ^r^ WHR- waist to hip ratio, ^s^ LMM-lean muscle mass, ^t^ TC- thigh circumference

^*^p < 0.05, **p < 0.01, ***p < 0.001

to convert kg to pound, multiply by 2.2

to convert cm to inches, multiply by 0.39

### Composition of the KD

Table 3 summarizes the composition of macronutrients of different diets as detailed in ten studies that reported calorie intake [12–14, 19, 25–27, 30, 33, 45].

**Table 3:** The composition of KD in studies (% EI)

KD composition (% EI)			
	CHO	Fat	Protein
Mean	15	60	25
Standard error	2.3	3.5	1
Median	10	63	24.7
SD	8.9	13.8	4
Sample variance	78.9	190.8	16.1
Range	22.5	44.2	12
Minimum	4.8	30.8	20
Maximum	27.3	75	32

In nine studies [8, 12, 14, 17, 24–26, 30, 45] participants in the KD were allowed ad libitum calorie consumption. In the study by Mohorko et al., [13], a calorie restriction of 1200–1500 kcal/day was implemented only during the first two weeks out of 12 weeks, with, EI unrestricted after week two. Similarly, participants in the study by Sethi et al., [27] were not required to count calories but were instructed to consume at least 1200 kcal/day. EI was not reported in six studies [8, 17, 24, 40, 46, 47], while the average calorie consumption in ten studies [12–14, 20, 25–27, 30, 33, 44] was 1634 kcal/day. None of the studies imposed a KD with an EI lower than 1200 kcal/day (maximum energy consumption was 2170 kcal/day) (Table 3).

### Effect of KD Intervention on Anthropometric Parameters

#### Weight Reduction

Most studies (14 out of 16, Table 2) demonstrated significant reductions in BW and body mass index (BMI) following a KD compared to control groups, usually calorie-restricted, low-fat diets (LFD). However, these reductions were less pronounced when the control diet closely mirrored the composition of the KD. For instance, Kikutchi et al., [24] observed substantial BW and BMI improvements in obese adults after a 2-month intervention with both an LCD and a very low-CHO diet (VLCD), with no significant differences between the two diets. Similarly, Guevara-Cruz et al., [47] didn't find significant differences between 3 groups of intervention (calorie-restricted diet, intermittent fasting, and KD) in all tested parameters: BW, BMI, WC, Visceral fat area (VFA), and BFP.

#### WC

In the study of Kikuchi et al.,[24], the Mediterranean diet (MD) and KD led to significant reductions in WC, but no statistical difference was found between the two diets. Similarly, Guevara-Cruz et al., [47] demonstrated a reduction in WC in three groups of intervention with no differences between the groups.

#### Lean Body Mass (LBM) and Skeletal Muscle Mass (SMM)

Mohorko et al., [13] noted a two-fold greater decrease in LBM among males compared to females. The loss of LBM continued progressively until week 12 only in males.

Paoli et al., [12] observed a reduction of less than 1 kg in absolute LBM; however, they reported an overall improvement in body composition, with a mean 7% increase in LBM percentage due to a significant FM loss. Valinejad and Khodaei[14] examined the role of physical activity across three groups following KD: KD alone, KD with aerobic training, and KD with resistance training. Their findings showed that combining KD with resistance training led to a 2.7 kg increase in LBM. In contrast, significant decreases in LBM were observed in the KD alone group (−1.3 kg) and the KD with aerobic training group (−1.7 kg)**.** Luong et al., [17] found a reduction of 1.4 kg of LBM only in the KD group. Perissiou et al**.,** [40]**,** measured the lean muscle mass (LMM) volume and reported a decrease of 854 ± 1670 cm^3^ after 8 weeks of KD + aerobic and resistance training. 4 studies [8, 27, 46, 47] measured SMM, with a combined mean reduction of 1.4 kg. Guevara-Cruz et el., [47] found an average increase of 1.1 kg in SMM after all interventions groups.

Table 4 summarizes the combined mean changes of major anthropometric outcomes of KD.

**Table 4:** Study Characteristics, Outcomes, and Methods

Parameter/Characteristic	Value
***Outcomes***	***Combined mean change (number of studies; duration)***
Body Weight (BW) (kg)	−7.9 (16 studies; 16.2 ± 11.8 weeks)
Body Mass Index (BMI) (kg/m^2^)	−3.2 (11 studies; 12.3 ± 6.9 weeks)
Waist Circumference (WC) (cm)	−7.2 (8 studies; 15.5 ± 14.5 weeks)
Visceral Adipose Tissue (VAT) (ml)	−2095.7 (3 studies; 17.3 ± 6.1 weeks)
Total Fat Mass (FM) (kg)	−8.2 (4 studies; 9.8 ± 4.5 weeks)
Body Fat Percentage (BFP) (%)	−3.5% (6 studies; 10.5 ± 7.2 weeks)
Waist-to-hip ratio (WHR)	−0.025 (3 studies; 10.7 ± 2.3 weeks)
Visceral Fat Area (VFA) (cm^2^)	−27.3 (3 studies; 10.7 ± 2.3 weeks)
Lean Body Mass (LBM) (kg)	−1.8 (6 studies; 14.5 ± 8.1 weeks)
Skeletal Muscle Mass (SMM) (kg)	−1.4 (4 studies; 15 ± 5.9 weeks)
***Study Characteristics***	
Follow-up period (mean)	16.2 weeks
Follow-up period (range)	3–52 weeks
Number of participants (mean)	63
Number of participants (range)	11–200
Frequency of meetings (mean)	Every 4.5 weeks
Frequency of meetings (range)	weekly to 6 times/year
***Methods for Measuring Ketones***	***Number of studies***
Urine	7 studies
Blood	8 studies
Breath	2 studies

^a^ Perissiou et al., [40] found a significant average reduction of 2.4 ± 5.7% in BFP after 8 weeks in two groups (KD and control) with no significant differences between the groups

^b^ Participants in the study by Michalczyk et al. [19] measured ketones in blood or urine

## Characteristics of the Studies

### Online vs Frontal Meeting

Most studies relied on in-person meetings and nutritional guidance provided by a qualified team [8, 12, 13, 17, 19, 24, 27, 30, 40, 45–48]. Pinsawas et al., [30] used both in-person and online meetings, and three studies [14, 25, 26] used only online meetings.

Table 4 summarizes some other characteristics of the studies:

### KD adherence -Methods used to Evaluate Adherence to the KD

Table 4 summarizes the methods used to measure ketone levels.

BK's were measured by researchers in eight studies [12, 13, 17, 24, 27, 30, 40, 47], while in two studies, participants self-measured breath acetone (BrAce) [25, 26], and in seven studies, participants used urine ketone sticks [8, 14, 19, 45–48]. Michalczyk et al., [19] used both blood and urine methods. Out of 16, only in nine studies [8, 12, 14, 17, 19, 24–26, 45], participants were in ketosis during the follow-up periods. Valinejad and Khodaei [14] excluded participants whose urine ketone levels were below 4 mmol/dl. Similarly, Luong et al., [17] excluded participants whose BK's were below 0.3 mmol/l. Sethi et al., [27] categorized participants as adherent if their BK levels were within 0.5–5 mmol/l for 80–100% of measurements, semi-adherent for 50–79% of measurements, and non-adherent if this threshold was achieved in less than 50% of measurements. Perissiou et al., [40] stratified participants based on the BK levels (BHB of 0.3 mmol/L ≥ achieved ketosis; BHB of 0.3 ≤ not in ketosis)**.** In the study of Pinsawas et al. [30], only 11% of the participants met the nutritional ketosis threshold of BHB > 0.5 mmol/L, whereas others met borderline criteria (4%) or remained below this level (85%)**.** Four studies [13, 46–48] did measure KB, however they didn't report the ketone levels of their participants. (Table 4).

### KD Adherence -Dietary Follow-Up

Different nutritional approaches were used to monitor nutritional adherence. Ten studies employed food record methodologies, four studies utilized a 3-day food record [13, 19, 30, 45], five studies [14, 24–26, 40] used 24-h dietary intake daily monitored by the research team, and Guevara-Cruz et al., [47] used both 24-h dietary intake and 3-day food record.

### The Impact of Ketone Levels on Weight-related Outcomes

Only three studies have directly examined the correlation between ketone levels or ketosis (BK levels typically between 0.5–5 mmol/L) and changes in body composition or weight loss. Falkenhain et al., [26], analyzed data from a digital KD intervention and observed that higher ketone levels were strongly correlated with greater weight loss. Sethi et al. [27], conducted a pilot trial in individuals with bipolar disorder and schizophrenia and found that participants who achieved consistent adherence to ketosis (ketone levels > 0.5 mmol/L for more than 80% of measurements) demonstrated significantly greater reductions in weight, FM, WC, and VAT compared to those with partial adherence (ketone levels > 0.5 mmol/L for 60–80% of measurements). Lastly, Perissiou et al., [40] investigated the impact of a LCD combined with exercise. They reported that achieving a ketogenic state (ketone levels > 0.3 mmol/L) was associated with a significant decrease in total BFP, VAT, and FMI. However, this study also noted a reduction in LMM during ketosis, highlighting a potential trade-off in body composition.

These findings collectively suggest that the degree and consistency of ketosis may influence the extent of weight and fat loss, with higher or more sustained ketone levels being associated with more pronounced changes. However, individual variability and potential impacts on LBM warrant further investigation.

### KD and Sex Differences

Only a few studies have examined the impact of sex on weight loss outcomes following a KD intervention. Mohorko et al., [13] reported significantly greater absolute weight loss in males compared to females (−18 ± 9 kg in males vs. −11 ± 3 kg in females). However, when weight loss was expressed as a percentage of baseline body weight, no significant sex differences were observed. Al Aamri et al., [8] found no significant differences in weight reduction between males and females following KD.

### The Impact of Physical Activity on Outcomes

Five studies [14, 40, 45, 46, 48] included physical activity in their intervention. Sun et al., [45] found that incorporated exercise training had no additional effects on weight loss. Li et al., [48] and Wu et al., [46] didn't analyze the impact of physical activity on anthropometric outcomes. Valinejad and Khodaei [14] found that resistance training positively affected LBM; participants implementing the KD with resistance training gained 2.66 kg while losing 5.6 kg during 6 weeks of intervention. In contrast, participants receiving only KD or even KD with aerobic training lost LBM. Perissiou et al., [40] gave their participants aerobic and resistance training and found that only the control group didn't lose LBM. Moreover, the reduction of FM wasn't statistically different between them and the ketogenic group**.**

### The Impact of Medical Treatment for Obesity

Wu et al., [46] found that participants receiving the GLP-1RA medication beinaglutide did not achieve statistically superior outcomes in BW, total FM, body fat, BFP, or VAT compared to those following a KD without medication. Similarly, Al Aamri et al. [8], reported no significant differences in mean weight reduction between participants using liraglutide and non-liraglutide users, regardless of whether they followed a VLCD or a low-calorie, low-fat control diet**.**

### Effect of the KD on Satiety

Only two studies [13, 14] assessed the impact of the KD on satiety. In the study by Mohorko et al., [13] participants on a 12-week KD experienced reduced appetite and emotional eating. Similarly, the study by Valinejad and Khodaei [14] found that combining exercise with a KD effectively altered appetite-regulating hormones and suppressed appetite sensation in overweight or obese men. These findings suggest that KDs, especially when combined with exercise, can positively influence appetite regulation and support weight management.

## Discussion

Current evidence supports the effectiveness of the KD for weight loss, demonstrating results comparable to pharmacological interventions. However, improper implementation can lead to risks, underscoring the importance of structured guidance. A notable limitation observed in several studies is the reduction in fat free mass (FFM), particularly among males or individuals following a KD without incorporating resistance training [8, 12–14, 17]. Ketosis, while promoting fat loss through water excretion and reduced insulin levels, may inhibit proteolysis, potentially contributing to a decrease in LMM [40].

Few studies have examined the effects of the KD on LBM. Among the available research, findings suggest that combining resistance training with a KD may mitigate reductions in LBM and could even promote increases in FFM. This emphasizes the need for individualized, structured guidance from healthcare professionals to optimize outcomes [6, 8, 23, 26, 49]. Alongside concerns about LMM loss, several studies documented mild or no side effects from the KD, such as headaches, fatigue, and gastrointestinal discomfort, mostly during the adaptation phase [8, 16, 24–27, 45, 46]. These findings highlight the importance of continuous monitoring and the development of strategies to minimize side effects, as they can influence adherence and patient safety. Standardizing weight loss indicators, such as WC, BFP, and VAT, is critical to ensure consistency across studies. These metrics offer valuable insights beyond BMI, with WC and VAT being particularly important due to their strong associations with metabolic and CVD risks [22, 28, 50]. WC measurement is especially practical, being simple, non-invasive, and widely accessible, making it suitable for use in both clinical and field settings as a predictor of abdominal fat and associated health risks.

Gender differences in response to the KD and weight loss remain an important area of investigation, considering the physical and biological disparities between men and women. Further research is needed to clarify these differences and refine dietary recommendations accordingly.

Adherence to the KD is often assessed using self-reported dietary records, which are prone to underreporting. Measuring ketone levels provides a more objective alternative but requires standardization across methods. While BK remains the gold standard, BrAce is emerging as a sensitive and practical alternative, especially at lower ketone levels where BrAce shows greater sensitivity to change than BK [2, 51]. Urine samples, though common, are not typically assessed quantitatively for acetoacetate, leading to significant measurement uncertainty [51].

The correlation between ketone levels and weight loss outcomes warrants further investigation, as higher BK levels have been linked to improved results in a limited number of studies [26, 27, 40].

The optimal CHO threshold and duration for maximizing benefits while maintaining adherence remain unresolved [1, 5, 6, 15, 23]. Despite its increasing popularity, many questions persist about the design, effectiveness, and long-term applicability of the KD for weight loss. Key challenges include small sample sizes, short intervention durations, and inconsistent definitions of ketosis and CHO thresholds in numerous studies [4, 6, 8, 10, 15, 16, 23, 28, 32, 34, 52]. Adherence to the KD, while comparable to other dietary approaches [23, 24, 53], is influenced by its restrictive nature and psychosocial factors, such as practical barriers to identifying compliant foods and limited social support [6, 23, 28]. Dietary satisfaction and perceived success in weight loss are critical factors for enhancing adherence, as they promote motivation and health awareness [7, 16, 24, 26, 36]. Developing tailored strategies to address psychosocial challenges and incorporating resistance training can mitigate risks such as FFM loss and improve outcomes.

KD can reduce appetite and improve eating behaviors, contributing to better adherence. Additionally, diet influences appetite-regulating hormones, which may further aid in weight management, especially when combined with resistance training. However, further research is needed to better understand the long-term effects of the KD on satiety and its role in sustaining weight loss.

### Limitations

Despite meeting the inclusion criteria for clinical intervention and ketone measurement, the cited studies exhibited significant variability in several key aspects. There was a lack of standardization in measuring ketones, leading to inconsistencies in reported outcomes. Additionally, the studies differed in their follow-up periods, dietary compositions, and intervention protocols, making direct comparisons challenging. This variability is not unique to the studies included in this review but reflects a broader limitation in KD research. Differences in study design, sample size, and measurement techniques are common, complicating the interpretation and generalizability of findings. Overall, these variations highlight the need for more standardized methodologies and consistent outcome measures to improve comparability and reliability in this field.

### Future Directions

Long-term studies are essential to evaluate the sustainability of the KD and its effects on weight loss maintenance, lean mass preservation, and metabolic health markers. Larger, long-term studies with standardized methodologies are needed to fully understand the potential of the KD. These studies should include comprehensive evaluations of key obesity markers, such as VAT and WC, as well as adherence assessments. Advances in ketone monitoring technology and individualized dietary prescriptions will be vital for refining the KD as a sustainable and effective weight loss strategy.

## Conclusion

When implemented correctly, the KD has significant potential as a weight-loss strategy. To achieve consistent and sustainable results, it is crucial to standardize dietary protocols, comprehensively monitor ketosis and body composition, and provide robust support mechanisms. Addressing the gaps identified in this review will optimize KD's application in clinical and research settings.

## Key References

• Unwin DJ, Tobin SD, Murray SW, Delon C, Brady AJ. Substantial and Sustained Improvements in Blood Pressure, Weight and Lipid Profiles from a Carbohydrate Restricted Diet: An Observational Study of Insulin Resistant Patients in Primary Care. Int J Environ Res Public Health. 2019 Jul 26;16(15):2680.
  - This study examines the long-term effects of a low-carbohydrate diet on patients with type 2 diabetes or impaired glucose tolerance in a primary care setting. Over an average duration of two years, the study found significant reductions in blood pressure, weight, and improvements in lipid profiles among participants adhering to the diet. Notably, these health benefits were achieved alongside a 20% reduction in antihypertensive medications, suggesting that carbohydrate restriction may be an effective and sustainable dietary intervention for managing insulin resistance and associated cardiovascular risk factors
• Goss AM, Gower B, Soleymani T, Stewart M, Pendergrass M, Lockhart M, et al. Effects of weight loss during a very low carbohydrate diet on specific adipose tissue depots and insulin sensitivity in older adults with obesity: a randomized clinical trial. Nutr Metab. 2020 Dec;17(1):64.
  - This study investigates the impact of a very low carbohydrate diet (VLCD) compared to a low-fat diet (LFD) on fat distribution and insulin sensitivity in older adults with obesity. The study found that participants on the VLCD experienced significantly greater reductions in total fat mass, particularly in visceral adipose tissue and intermuscular adipose tissue, and improvements in insulin sensitivity compared to those on the LFD. These findings suggest that VLCDs may be more effective than LFDs in reducing metabolically harmful fat depots and enhancing insulin sensitivity in this population.
• Paoli A, Bianco A, Moro T, Mota JF, Coelho-Ravagnani CDF. The Effects of Ketogenic Diet on Insulin Sensitivity and Weight Loss, Which Came First: The Chicken or the Egg? Nutrients. 2023 Jul 12;15(14):3120.
  - This review examines the dual impact of ketogenic diets (KDs) on weight loss and insulin sensitivity. It discusses whether improvements in insulin sensitivity are a direct result of the KD or a consequence of weight loss induced by the diet. The article delves into the biochemical mechanisms underlying these effects, providing a comprehensive analysis of how KDs influence glycemic control and metabolic health.
• Kolb H, Kempf K, Röhling M, Lenzen-Schulte M, Schloot NC, Martin S. Ketone bodies: from enemy to friend and guardian angel. BMC Med. 2021 Dec 9;19(1):313.
  - This review explores the evolving understanding of ketone bodies beyond their traditional role as alternative energy sources during carbohydrate scarcity. The authors discuss how ketone bodies not only serve as fuel but also promote resistance to oxidative and inflammatory stress, and there is a decrease in anabolic insulin-dependent energy expenditure. This perspective highlights the potential therapeutic applications of ketone bodies in managing conditions such as obesity, type 2 diabetes, and cardiovascular diseases.

## Data Availability

No datasets were generated or analysed during the current study.
